# Supplementary material for: Average egg price: Mean reversion and persistence in a time series approach
Source: PLoS One. 2026 Mar 25;21(3):e0337054. doi: 10.1371/journal.pone.0337054 (PMC13016358; doi:10.1371/journal.pone.0337054)
Supplement: Attachment — Submitted filename: PLOSONE.pdf [file pone.0337054.s003.pdf]

Review Report for Manuscript PONE-D-25-28658

"Average Egg Price: Mean Reversion, Persistence and Structural Breaks"

Overview

Strengths of the manuscript:

- The paper tackles a relevant topic (egg prices, inflation, and persistence).
- It attempted to uses sound econometric techniques (fractional integration and FC-VAR).
- Structure is coherent: Introduction, Literature Review, Methodology, Empirical Results, Conclusion.
- Data sources and motivation are clearly presented.

However, this report provides a detailed review of the manuscript, focusing on two key aspects:

- Grammatical and stylistic revisions
- Technical accuracy and methodological suggestions

Comments are anchored with reference to specific line numbers for clarity and actionable feedback. In addition, the manuscript is attached to this report and annotated with two colors; orange and blue respectively denoting grammar and technical issues.

1. Grammatical and Style Suggestions

- **Lines 15–17:** Replace "long term" with **"long-term"** (hyphenated compound adjective).
- **Line 17:** Change "exhibits traits of long memory or mean reversion" to **"exhibits evidence of long memory or mean reversion"**.
- **Line 19:** Add commas to "i.e." **"i.e., I(0) stationarity"**.
- **Lines 91–92:** Rephrase "will be elaborated on" as **"will be provided throughout the paper"**.

- **Line 121:** Improve clarity: "grasp the data properties..."
  "understand the data properties and assess whether the findings are consistent".
- **Line 188:** Simplify "in order to attempt to forecast" to **"to forecast"**.
- **Line 295:** Reword "intermediate degree of differentiation below 1" as
  "fractional degrees of differencing below 1".
- **Article and Capitalization Corrections:**
  - "cost per Dozen" → "cost per dozen"

2. Abstract Revision Suggestion

Original:

> In this paper, we aim to analyze the long term behavior of the average egg price...

Improved Version:

> This study investigates the long-term behavior of average egg prices in the U.S., using fractional integration techniques to assess persistence and mean reversion. The analysis also explores the relationship between egg prices and the Producer Price Index (PPI) through cointegration methods. The findings suggest a lack of short memory in both series, although mean reversion is observed for the PPI. Additionally, the FCVAR model indicates a potential long-run equilibrium relationship between average egg prices and the PPI.

3. Technical Issues and Methodological Enhancements

3.1 Technical & Analytical Concerns

a. Misinterpretation Risk:

The claim mean reversion takes place for the PPI data needs clarity. The value of d between 0.5 and 1 implies nonstationary but mean-reverting behavior, not strong reversion like I(0).

b. FCVAR Interpretation Caution Needed:

- The authors state: cointegrating errors are I(0) from $d = b = 0.723 \Rightarrow$ implies no reduction in integration order, contradicting Engle-Granger's cointegration concept which expects $d > b$.
- Silent Issue: If $d = b$, then no cointegration under strict definition (i.e., no reduction in integration order). The conclusion of "stable long-run equilibrium" may be overstated or needs qualification.

c. Breitung and Candelon Test Table Interpretation:

Ensure to emphasize that "Average Egg Price → PPI is only significant in short and medium term – not long term. This undermines symmetry and may impact the cointegration claim.

3.2 Structural Issues

a. Literature Review:

- Too brief. Suggest expanding with more recent papers on fractional integration and food price volatility, including:
- Recent inflation studies (post-2020 pandemic).
- Literature on commodity price shocks and persistence.

b. Discussion Section:

The conclusions are descriptive rather than critical analysis. Recommend adding:

- Implications for policy – what should policymakers do?
- Limitations – e.g., model assumptions, data constraints.

Mathematical presentation of the manuscript technical salient issues follows:

1. Spectral Density Definition Error (Lines 249–254)

The manuscript defines:

$$f(\lambda) = \sum_{j=-\infty}^{\infty} \gamma(u) e^{i\lambda u} = \sum_{j=-\infty}^{\infty} \gamma(u) \cos(\lambda u)$$

Issues:

1. **Index Mismatch:** The summation uses index j, but $\gamma(u)$ remains inconsistent.
2. **Incorrect Equality:** The cosine form represents only the real part. The correct expressions are:

$$f(\lambda) = \frac{1}{2\pi} \sum_{j=-\infty}^{\infty} \gamma(j) e^{-i\lambda j}, \quad \text{or} \quad f(\lambda) = \frac{1}{2\pi} \left[\gamma(0) + 2 \sum_{j=1}^{\infty} \gamma(j) \cos(\lambda j) \right]$$

2 Methodological Robustness (Lines 198, 295, 343)

The justification for fractional integration is inadequately presented in the study, and robustness of the estimation approach could be improved.

Suggestions:

- **Line 198:** Introduce alternative long-memory estimators, e.g., Local Whittle (Robinson, 1995), log-periodogram (Geweke & Porter-Hudak, 1983), and wavelet-based methods (Abry & Veitch, 1998).
- **Line 295:** Note that FDML relies on parametric assumptions. Cross-validation with other estimators is advisable.
- **Line 343:** Suggest robustness checks with semi-parametric methods to confirm persistence properties.

3. FCVAR Interpretation Caveat (Lines 417–419)

The reported FCVAR result shows $d = b = 0.723$. However, fractional cointegration typically requires $d > b$ for mean-reverting cointegrating relationships. If $d = b$, the residuals are $I(d)$ and may not revert to equilibrium, undermining the interpretation of long-run stability. This interpretation should be revised or qualified.

4. Suggested References for Inclusion

- Robinson, P.M. (1995). Gaussian semi-parametric estimation of long range dependence. *Annals of Statistics*, **23**(5), 1630–1661.
- Geweke, J., & Porter-Hudak, S. (1983). The estimation and application of long memory time series models. *Journal of Time Series Analysis*, **4**(4), 221–238.
- Abry, P., & Veitch, D. (1998). Wavelet analysis of long-range-dependent traffic. *IEEE Transactions on Information Theory*, **44**(1), 2–15.

Manuscript Reference Citation and List

Line 185-186: Raghavender and Udayshankar (2020) is cited but not listed on the reference list.

4. Conclusion

The manuscript presents an important and timely economic study. However, important technical and interpretation issues must be addressed before it can be recommended for publication. Revisions are needed to correct mathematical inaccuracies, strengthen methodological arguments, improve clarity, and align conclusions with the evidence.

Recommendation: Major Revision. Additional review comments are provided in the attached annotated PDF.
