# Supplementary material for: Average egg price: Mean reversion and persistence in a time series approach
Source: PLoS One. 2026 Mar 25;21(3):e0337054. doi: 10.1371/journal.pone.0337054 (PMC13016358; doi:10.1371/journal.pone.0337054)
Supplement: Attachment — Submitted filename: Report new.docx [file pone.0337054.s004.docx]

# Reviewer Report

Manuscript Title: Average Egg Price: Mean reversion, Persistence and Structural Breaks

Journal: PLOS ONE

This paper offers a methodologically sound and policy-relevant contribution to price dynamics in the agricultural sector. With minor revisions to include more economic interpretation, visual data summaries, and connections to recent literature, it could serve as a valuable reference for both academic and applied economists.

1) The econometric results are robust, further interpretation of what the lack of mean reversion implies for policy (e.g., subsidy design or inventory stabilization) would enhance the manuscript’s impact.
2) You need to add the graphical visualization like time series plots, seasonal decomposition, and residual diagnostics are missing, these would help readers better understand the data characteristics and model fit.
3) Terms like "explosive pattern" and "mean reversion" should be more explicitly linked to economic behavior and policy interpretation.
4) The Federal Reserve and BLS are mentioned, include the exact FRED series codes or links for reproducibility.
5) Line 208–209: Consider clarifying the direct link between egg price peaks and the HPAI outbreak with reference to supply constraints or market responses.
6) doi: <https://doi.org/10.1016/j.najef.2022.101766>; doi:10.1080/07350015.2023.2277172
