# Supplementary material for: Average egg price: Mean reversion and persistence in a time series approach
Source: PLoS One. 2026 Mar 25;21(3):e0337054. doi: 10.1371/journal.pone.0337054 (PMC13016358; doi:10.1371/journal.pone.0337054)
Supplement: Attachment — Submitted filename: LETTER ADDRESSING THE CHANGES.docx [file pone.0337054.s005.docx]

**Plos One**	Prof. Luis A. Gil-Alana

Muhammad Umer Arshad	University of Navarra

Academic Editor	Pamplona, Spain

July 7^th^, 2025

**Ms.Ref. No.:**	PONE-D-25-28658

**Title:**	Average Egg Price: Mean reversion, Persistence and Structural Breaks

**Journal:**	PLOS ONE

Dear Editor,

Many thanks for considering our paper entitled “Average Egg Price: Mean reversion,

Persistence and Structural Breaks” as a candidate for publication in Plos One.

Following your advice and the reviewers’ comments, we have made some

additional changes in the paper that we summarize in the following pages. Hoping this

time the paper might be acceptable for publication in PONE,

Yours sincerely,

Prof. Luis A. Gil-Alana

Yours sincerely

Prof. Luis A. Gil-Alana

University of Navarra

Pamplona

Spain

P.S.:	The funders had no role in study design, data collection and analysis, decision to publish, or preparation of the manuscript.

**Journal requirements:**

When submitting your revision, we need you to address these additional requirements.

**1.)**	Please ensure that your manuscript meets PLOS ONE's style requirements, including those for file naming. The PLOS ONE style templates can be found at

<https://journals.plos.org/plosone/s/file?id=wjVg/PLOSOne_formatting_sample_main_body.pdf> and

[https://journals.plos.org/plosone/s/file?id=ba62/PLOSOne_formatting_sample_title_authors_affiliations.pdf](https://track.editorialmanager.com/CL0/https:%2F%2Fjournals.plos.org%2Fplosone%2Fs%2Ffile%3Fid=ba62%2FPLOSOne_formatting_sample_title_authors_affiliations.pdf/1/010f0197cbfc9c40-d765f0b0-29e8-4946-a5e0-1d6de56a863d-000000/KJwmrgCid1DDcsE-UMLBQHL_AVOm6pbaBZPbtqUWnFM=217)

Done.

**2.)**	Thank you for stating the following financial disclosure:

 [Prof. Luis A. Gil-Alana gratefully acknowledges financial support from the PID2023-149516NB-I00/ AEI/10.13039/501100011033/ FEDER, UE project from ‘Ministerio de Economía, Industria y Competitividad’ (MINEIC), ‘Agencia Estatal de Investigación’ (AEI) Spain and ‘Fondo Europeo de Desarrollo Regional’ (FEDER), and also from Internal Projects of the Universidad Francisco de Vitoria.].

Please state what role the funders took in the study.  If the funders had no role, please state: "The funders had no role in study design, data collection and analysis, decision to publish, or preparation of the manuscript."

If this statement is not correct you must amend it as needed.

Please include this amended Role of Funder statement in your cover letter; we will change the online submission form on your behalf.

Done.

**3.)**	In the online submission form, you indicated that [The data are available from the authors upon request.].

All PLOS journals now require all data underlying the findings described in their manuscript to be freely available to other researchers, either 1. In a public repository, 2. Within the manuscript itself, or 3. Uploaded as supplementary information.

This policy applies to all data except where public deposition would breach compliance with the protocol approved by your research ethics board. If your data cannot be made publicly available for ethical or legal reasons (e.g., public availability would compromise patient privacy), please explain your reasons on resubmission and your exemption request will be escalated for approval.

Data are available in a supplementary file (DATA FILE.XLS).

**Reviewers' comments:

Reviewer's Responses to Questions**

**Comments to the Author
1.)**	Is the manuscript technically sound, and do the data support the conclusions?
Reviewer #1: Partly

Reviewer #2: Yes

**2.)**	Has the statistical analysis been performed appropriately and rigorously?

Reviewer #1: No

Reviewer #2: Yes

**3.)**	Have the authors made all data underlying the findings in their manuscript fully available?
Reviewer #1: Yes

Reviewer #2: Yes

**4.)**	Is the manuscript presented in an intelligible fashion and written in standard English?
Reviewer #1: No

Reviewer #2: Yes

**5.)**	Review Comments to the Author
Reviewer #1: The manuscript presents an important and timely economic study. However, important technical and interpretation issues must be addressed before it can be recommended for publication. Revisions are needed to correct mathematical inaccuracies, strengthen methodological arguments, improve clarity, and align conclusions with the evidence.
Recommendation: Major Revision. Additional review comments are provided in the attached annotated PDF.

Reviewer #2: This paper offers a methodologically sound and policy-relevant contribution to price dynamics in the agricultural sector. With minor revisions to include more economic interpretation, visual data summaries, and connections to recent literature, it could serve as a valuable reference for both academic and applied economists.

**6.)**	PLOS authors have the option to publish the peer review history of their article ([what does this mean?](https://track.editorialmanager.com/CL0/https:%2F%2Fjournals.plos.org%2Fplosone%2Fs%2Feditorial-and-peer-review-process%23loc-peer-review-history/1/010f0197cbfc9c40-d765f0b0-29e8-4946-a5e0-1d6de56a863d-000000/ZCFrm62B8bPwCCQjNxnsaILRMQCZyBRbmRhYfmm65JU=217)). If published, this will include your full peer review and any attached files.
**Do you want your identity to be public for this peer review?** For information about this choice, including consent withdrawal, please see our [Privacy Policy](https://track.editorialmanager.com/CL0/https:%2F%2Fwww.plos.org%2Fprivacy-policy/1/010f0197cbfc9c40-d765f0b0-29e8-4946-a5e0-1d6de56a863d-000000/3R8B3LvxZoOShuuLpBRZAOWnRRaGF7HevURA7mf39S4=217).

Reviewer #1: **Yes:** Kazeem Babatunde Akande

Reviewer #2: No

**Review Report for Manuscript PONE-D-25-28658**

**Average Egg Price: Mean Reversion, Persistence and Structural Breaks"**

**Overview**

**Strengths of the manuscript:**

_ The paper tackles a relevant topic (egg prices, inflation, and persistence).

_ It attempted to uses sound econometric techniques (fractional integration and FC-

VAR).

_ Structure is coherent: Introduction, Literature Review, Methodology, Empirical Re-

sults, Conclusion.

_ Data sources and motivation are clearly presented.

However, this report provides a detailed review of the manuscript, focusing on two key

aspects:

_ Grammatical and stylistic revisions

_ Technical accuracy and methodological suggestions

Comments are anchored with reference to specific line numbers for clarity and actionable feedback. In addition, the manuscript is attached to this report and annotated with two colors; orange and blue respectively denoting grammar and technical issues.

**Reply:**	Many thanks for the effort made for this revision.

**1. Grammatical and Style Suggestions**

_ Lines 15-17: Replace “long term" with \long-term" (hyphenated compound adjec-

tive).

Done.

_ Line 17: Change “exhibits traits of long memory or mean reversion" to

“exhibits evidence of long memory or mean reversion".

Done.

_ Line 19: Add commas to “i.e." \i.e., I(0) stationarity".

Done.

_ Lines 91{92: Rephrase “will be elaborated on" as

\will be provided throughout the paper".

Done.

_ Line 121: Improve clarity: “grasp the data properties..."

“understand the data properties and assess whether the findings are con-

sistent".

Done.

_ Line 188: Simplify “in order to attempt to forecast" to “to forecast".

Done.

_ Line 295: Reword “intermediate degree of differentiation below 1" as

“fractional degrees of differencing below 1".

Done.

_ Article and Capitalization Corrections:

{ “cost per Dozen" by “cost per dozen"}.

Done.

**2. Abstract Revision Suggestion**

Original:

In this paper, we aim to analyze the long term behavior of the average egg price...

Improved Version:

This study investigates the long-term behavior of average egg prices in the U.S.,

using fractional integration techniques to assess persistence and mean reversion.

The analysis also explores the relationship between egg prices and the Producer

Price Index (PPI) through cointegration methods. The findings suggest a lack

of short memory in both series, although mean reversion is observed for the

PPI. Additionally, the FCVAR model indicates a potential long-run equilibrium

relationship between average egg prices and the PPI.

**Reply:**	Many thanks. The new Abstract is more focus and clearer than the one presented in the old version. Thanks again.

**3. Technical Issues and Methodological Enhancements**

**3.1 Technical & Analytical Concerns**

**a. Misinterpretation Risk:**

The claim mean reversion takes place for the PPI data needs clarity. The value of

d between 0.5 and 1 implies nonstationary but mean-reverting behavior, not strong

reversion like I(0).

**Reply:**	Thanks. We have improved this section by adding the following:

“ *… The estimates of d are equal to 0.71 (white noise), 0.69 (Bloomfield) and 0.70 (seasonal AR) for the original data, and for the logged data, the values are 0.65 (white noise and seasonal AR) and 0.71 with Bloomfield disturbances. The fact that the orders of integration are in the interval [0.5, 1) indicate that the process of reversion is slow compared with the standard case of I(0) errors.*

*Based on the estimated parameters, it is reasonable to conclude that fluctuations in average egg prices will have long lasting effects in the U.S., as none of the series display mean reversion, while changes in the Production Price Index tend to return to the long-term average over time, though the process is relatively slow. …*”.

**b. FCVAR Interpretation Caution Needed:**

_ The authors state: cointegrating errors are I(0) from d = b = 0:723 ) implies

no reduction in integration order, contradicting Engle-Granger's cointegration

concept which expects d > b.

_ Silent Issue: If d = b, then no cointegration under strict definition (i.e., no reduc-

tion in integration order). The conclusion of "stable long-run equilibrium" may

be overstated or needs qualification.

**Reply:** Thanks for the comment. We have clarified this point that was unclear in the old version. In fact, it contained an error in the interpretation. Note that d refers to the order of integration of the individual series, while b is the magnitude of the reduction in the order of integration in the cointegrating errors. In other words, the degree of integration in the cointegrating errors is d – b. Thus, the fact that d = b implies that the errors are I(0) and cointegration exists between the variables.

We have explained this point further in the following lines:

“ *… To grasp the findings accurately, it is essential to consider the definition of cointegration in a bivariate context as outlined by Engle and Granger (1987). They define cointegration as a situation where the two individual series are integrated of order d (i.e., I(d) ), but there exists a linear combination of the two that is integrated of a smaller order, denoted as d - b, where b > 0.*

*Upon examining the integrating and cointegrating parameters, it becomes apparent that for both scenarios, the order of integration for each series and the reduction in the order in the cointegrating regression stand at 0.723 (d = b = 0.723). These results imply I(0) cointegrating errors, as there is a relationship between the two series of order 0.723 - 0.723 = 0. Such results indicate that there is evidence of a stable long-run equilibrium relationship between the Average Egg price and the Producer Price Index in the U.S., implying long run co-movement between the two variables. …*”.

**c. Breitung and Candelon Test Table Interpretation:**

Ensure to emphasize that "Average Egg Price → PPI is only significant in short and

medium term not long term. This undermines symmetry and may impact the coin-

tegration claim.

**Reply:**	Thanks for the comment, which has been incorporated in the conclusions:

“ *… Nevertheless, it should be taken into account that this relationship (Average Egg Price → PPI) is only significant in the short and medium term but not in the long term, which may be undermining symmetry and the cointegration relationship. …*”.

**3.2 Structural Issues**

**a. Literature Review:**

_ Too brief. Suggest expanding with more recent papers on fractional integration

and food price volatility, including:

_ Recent inflation studies (post-2020 pandemic).

_ Literature on commodity price shocks and persistence.

We have included some papers using fractionally integrated methods on inflation rates as well as others relating commodity prices with persistence:

*“ … Thus, in the former case mean reversion takes place and shocks will tend to disappear by themselves in the long run implying lack of policy actions to recover the original level of prices. On the other hand, on the presence of unit roots or explosive patterns shocks will have a permanent nature and strong policy actions will be required to recover the original prices.* *Note that the main advantage of the fractional framework is that it allows any real value for the degree of differentiation including then the particular cases of stationarity I(0) and nonstationarity I(1) as particular cases of interest of our approach. Recent examples of empirical applications of fractional integration on inflation rates include among others the paper by Usman and Gil-Alana (2025) and Solarin et al. (2025) and other applications dealing with persistence in commodity prices are Alfeus and Nikitopoulos (2022), Gil-Alana and Font de Villanueva (2022) and Basistha and Startz (2024) …”.*

b. Discussion Section:

The conclusions are descriptive rather than critical analysis. Recommend adding:

_ Implications for policy { what should policymakers do?

_ Limitations { e.g., model assumptions, data constraints.

**Reply:** Thanks for these suggestions. With respect to the policy implications, we have added the following comment on Section 5 (Policy implications and conclusions)

“ *… With regards to the average egg price, the unit root null hypothesis, i.e., d = 1 is rejected under the white noise and seasonal AR models as d is much higher than 1, indicating explosive patterns, whereas for the Bloomfield (1973) model such hypothesis cannot be rejected. Nevertheless, in both cases we observe lack of reversion to the mean, implying permanency of shocks and requiring policy actions (e.g., subsidy design or inventory stabilization) in case of shocks to recover the original trends. Conversely, for the Producer Price index data, mean reversion seems to take place in all cases, making it useless for policymakers to take action when sudden peaks occur. …”.*

Limitations of the present work and lines of future research:

“ *… The paper contains some limitations. First, the possibility of structural breaks is an issue that has not been investigated in the present work. This is relevant since long memory and fractional integration are closely related to the issue of breaks. In addition, non-linear trends can replace the linear structure employed in this work. Other alternative long memory model can also be used. …*”.

Mathematical presentation of the manuscript technical salient issues follows:

1. Spectral Density Definition Error (Lines 249{254)

The manuscript defines:

$f\left( \lambda\right)=\sum_{j=-\infty}^{\infty} \gamma\left( u \right)e^{i \lambda u} = \sum_{j=-\infty}^{\infty} \gamma\left( u \right)coscos \left( \lambda u \right) .$

**Reply:**	Thanks for the comment. The formula for the spectral density function has been corrected in the current version:

$f\left( \lambda\right)=\frac{1}{2\pi}\sum_{j=-\infty}^{\infty} \gamma\left( j \right)e^{i \lambda j} = \frac{1}{2\pi} \left( \gamma\left( 0 \right) + 2 \sum_{j=1}^{\infty} \gamma\left( j \right) cos \left( \lambda j \right) \right).$

**Issues:**

2. Index Mismatch: The summation uses index j, but (u) remains inconsistent.

Corrected.

2. Incorrect Equality: The cosine form represents only the real part. The correct expressions are

$f\left( \lambda\right)=\frac{1}{2\pi}\sum_{j=-\infty}^{\infty} \gamma\left( j \right)e^{i \lambda j} = \frac{1}{2\pi} \left( \gamma\left( 0 \right) + 2 \sum_{j=1}^{\infty} \gamma\left( j \right) cos \left( \lambda j \right) \right).$

Corrected.

**2 Methodological Robustness (Lines 198, 295, 343)**

The justification for fractional integration is inadequately presented in the study, and robustness of the estimation approach could be improved.

**Reply:**	Thanks. We have added first that:

*“ … Note that the main advantage of the fractional integration framework is that it allows any real value for the degree of differentiation including then the particular cases of stationarity I(0) and nonstationarity I(1) as particular cases of interest of our approach. …”.*

**Suggestions:**

_ Line 198: Introduce alternative long-memory estimators, e.g., Local Whittle (Robin-

son, 1995), log-periodogram (Geweke & Porter-Hudak, 1983), and wavelet-based methods (Abry & Veitch, 1998).

As a robustness check, we have added the following in the methodological section:

“ *… The estimation relies on Robinson (1994), which is a testing procedure based on the Lagrange Multiplier (LM) principle and that uses the likelihood function in the frequency domain. We use a very simple approach of this method that has been widely used in the empirical literature (e.g., Gil-Alana and Robinson, 1997). The main features of this procedure are that it is valid for any real d and thus it does not impose preliminary differentiation in case of nonstationary data; moreover, it has a standard normal limit distribution and it is the most efficient method in the context of local alternatives. Nevertheless, as a robustness check, we also employed alternative approaches like the parametric method of Sowell (1992) and the semiparametric log-periodogram estimate of Geweke and Porter-Hudak (1983), both producing almost identical results to those reported in this work. …*”.

_ Line 295: Note that FDML relies on parametric assumptions. Cross-validation with

other estimators is advisable.

_ Line 343: Suggest robustness checks with semi-parametric methods to confirm persistence properties.

**Reply:**	Thanks for the comment. Though our method is fully parametric, we also employed semiparametric approaches like the log-periodogram estimate of Geweke and Porter-Hudak (GPH, 1983), improved later in Robinson (1995a) and Doukhan et al. (2003) and the local-Whittle approach of Robinson (1995b) improved by Shimotsu and Phillips (2005) among others. (See the previous comment).

**3. FCVAR Interpretation Caveat (Lines 417{419)**

The reported FCVAR result shows d = b = 0:723. However, fractional cointegration typically requires d > b for mean-reverting cointegrating relationships. If d = b, the residuals are I(d) and may not revert to equilibrium, undermining the interpretation of long-run stability. This interpretation should be revised or qualified.

**Reply:**	Many thanks for this very valuable comment. We have clarified this point earlier, clearly stating that d indicates the order of integration of the individual series, while d – b refers to the degree of integration of the cointegrating errors; thus, if d = b, the cointegrating errors are stationary I(0) and cointegration holds if d > 0. See the comment above.

**4. Suggested References for Inclusion**

_ Robinson, P.M. (1995). Gaussian semi-parametric estimation of long range depen-

dence. Annals of Statistics, 23(5), 1630{1661.

_ Geweke, J., & Porter-Hudak, S. (1983). The estimation and application of long

Memory time series models. Journal of Time Series Analysis, 4(4), 221{238.

_ Abry, P., & Veitch, D. (1998). Wavelet analysis of long-range-dependent traffic. IEEE Transactions on Information Theory, 44(1), 2{15.

**Manuscript Reference Citation and List**

Line 185-186: Raghavender and Udayshankar (2020) is cited but not listed on the reference list.

**Reply:**	Thanks. We have included the above references, including Raghavender and Udayshankar (2020):

Raghavender, M. and M. Udayshankar (2020), Forecasting of egg prices in Telangana using R-Software, International Journal of Approximate Reasoning 10(3).

**4. Conclusion**

The manuscript presents an important and timely economic study. However, important technical and interpretation issues must be addressed before it can be recommended for publication. Revisions are needed to correct mathematical inaccuracies, strengthen methodological arguments, improve clarity, and align conclusions with the evidence.

**Recommendation:** Major Revision. Additional review comments are provided in the

attached annotated PDF.

**Reply:**	Thanks for all these positive comments and for the annotated PDF file, which has been extremely useful for the improvement of the manuscript.

**Other minor changes:**

Title page:	Comments from the Editor and two anonymous reviewers are gratefully acknowledged.

We have corrected some other numerous typos and grammatical errors all over the manuscript.

**References**

Abry, P., and D. Veitch (1998). Wavelet analysis of long-range-dependent traffic. IEEE Transactions on Information Theory, 44(1), 2{15.

## Alfeus, M. and C.S. Nikitopoulos (2022), Forecasting volatility in commodity markets with long-memory models, [Journal of Commodity Markets](https://www.sciencedirect.com/journal/journal-of-commodity-markets) 28, 100248

Basisstha, A. and R. Startz, (2024), Measuring persistent global economic factors with output, commodity price, and commodity currency data, Journal of Forecasting 43(7), 2860-2885.

Bloomfield, P. (1973). An exponential model in the spectrum of a scalar time series, Biometrika 60, 217-226.

Breitung, J. and B. Candelon (2006). “Testing for short- and long-run causality: A frequency-domain approach”. Journal of Econometrics [online], pp 363-368.

Doukhan, P., Oppenheim, G., & Taqqu, M. (2003). Theory and Applications of Long-Range Dependence. Birkhauser.

Engle, R. and Granger, C.W.J. (1987). Cointegration and error correction. Representation, estimation and testing. Econometrica 55: 251-276.

Geweke, J. & Porter-Hudak, S. (1983), The estimation and application of long memory time series. *Journal of Time Series Analysis 4*(4), 221-238. https://doi.org/10.1111/j.1467-9892.1983.tb00371.x

Gil-Alana, L.A. and P.M. Robinson, (1997). Testing of unit root and other nonstationary hypotheses in macroeconomic time series. Journal of Econometrics, 80(2), 241-268. doi:10.1016/s0304-4076(97)00038-9

Raghavender, M. and M. Udayshankar (2020), Forecasting of egg prices in Telangana using R-Software, International Journal of Approximate Reasoning 10(3).

Robinson, P. M. (1994). Efficient Tests of Nonstationary Hypotheses. Journal of the American Statistical Association, 89(428), 1420–1437. <https://doi.org/10.2307/2291004>

Robinson, P. M. (1995a). Log-periodogram regression of time series with long range dependence. Annals of Statistics, 23, 1048–1072.

Robinson, P. M. (1995b). Gaussian semiparametric estimation of long-range dependence. *Annals of Statistics 23,* 1630–1661.

Shimotsu, K. and P.C.B. Phillips, (2005), Exact local Whittle estimation of fractional integration, Annals of Statistics 33(4),

18901933. https://doi.org/10.1214/009053605000000309

Sowell, F. (1992), Maximum likelihood estimation of stationary univariate fractionally integrated time series models, *Journal of Econometrics 53*(1-3), 165-188. https://doi.org/10.1016/0304-4076(92)90084-5

# Reviewer Report

**Manuscript Title: Average Egg Price: Mean reversion, Persistence and Structural Breaks**

**Journal: PLOS ONE**

This paper offers a methodologically sound and policy-relevant contribution to price dynamics in the agricultural sector. With **minor revisions** to include more economic interpretation, visual data summaries, and connections to recent literature, it could serve as a valuable reference for both academic and applied economists.

**1.)**	The econometric results are robust, further interpretation of what the lack of mean reversion implies for policy (e.g., subsidy design or inventory stabilization) would enhance the manuscript’s impact.

**Reply:**	Many thanks for this comment. We have added the following paragraph in the Conclusions in the new version of the paper:

“ *… With regards to the average egg price, the unit root null hypothesis, i.e., d = 1 is rejected under the white noise and seasonal AR models as d is much higher than 1, indicating explosive patterns, whereas for the Bloomfield (1973) model such hypothesis cannot be rejected. Nevertheless, in both cases we observe lack of reversion to the mean, implying permanency of shocks and requiring policy actions (e.g., subsidy design or inventory stabilization) in case of shocks to recover the original trends. Conversely, for the Producer Price index data, mean reversion seems to take place in all cases, making it useless for policymakers to take action when sudden peaks occur. …*”.

**2.)**	You need to add the graphical visualization like time series plots, seasonal decomposition, and residual diagnostics are missing, these would help readers better understand the data characteristics and model fit.

**Reply:**	Thanks. We have included a figure with the time series plot of the data (Figure 2). Dealing with the residual diagnostic, this is another interesting issue and we have further explained it in the following way. We first have presented in a clearer way the methodology used, stressing its efficiency against local departures:

“ *…*	*The estimation relies on Robinson (1994), which is a testing procedure based on the Lagrange Multiplier (LM) principle and that uses the likelihood function in the frequency domain. We use a very simple approach of this method that has been widely used in the empirical literature (e.g., Gil-Alana and Robinson, 1997). The main features of this procedure are that it is valid for any real d and thus it does not impose preliminary differentiation in case of nonstationary data; moreover, it has a standard normal limit distribution and it is the most efficient method in the context of local alternatives. Nevertheless, as a robustness check, we also employed alternative approaches like the parametric approach of Sowell (1992) and the semiparametric log-periodogram estimate of Geweke and Porter-Hudak (1983), both producing almost identical results to those reported in this work. …*”.

Dealing with the diagnostic check, we have added the following comment:

*“ … Though not reported in the manuscript, we also conducted some diagnostic check on the estimated residuals on the d-differenced series; in particular, we implemented various Portmanteau tests* *(Box and Pierce, 1970; Ljung and Box, 1978) and the Lagrange Multiplier (LM) test of Breusch and Godfrey (Breusch, 1978; Godfrey, 1978), and the results reported evidence of no additional serial correlation in the data. …”.*

**3.)**	Terms like "explosive pattern" and "mean reversion" should be more explicitly linked to economic behavior and policy interpretation.

**Reply:**	Thanks. See the following comment added at the end of Section 2:

“ *… Moreover, the fractionally integrated or I(d) models can help us discern whether shocks in the series have transitory, either with exponentially fast decays (i.e., I(0)) or hyperbolically slow decays (I(d), 0 < d < 1) versus permanent or even explosive patterns (I(d), d ≥ 1) (Gil-Alana, Mudida and Zerbo, 2021). Thus, in the former case mean reversion takes place and shocks will tend to disappear by themselves in the long run implying lack of policy actions to recover the original level of prices. On the other hand, on the presence of unit roots or explosive patterns shocks will have a permanent nature and strong policy actions will be required to recover the original prices. …*”.

**4.)**	The Federal Reserve and BLS are mentioned, include the exact FRED series codes or links for reproducibility.

**Reply:**	This is now done in the endnote of Figures 1 and 2.

**5.)**	Line 208–209: Consider clarifying the direct link between egg price peaks and the HPAI outbreak with reference to supply constraints or market responses.

**Reply:**	Thanks. We have added the following comment:

*“ …* *We observe that the highest average egg price was recorded in 2023-01-01, 11 months after the first reported HPAI in the U.S. The HPAI devastated poultry flocks across the country, pushing egg prices to record highs, an issue exacerbated by inflation and persistent supply chain disruptions. During this period, egg production measured by the dozen decreased by 6.45%, dropping from 806,416,200 in January 2022 to 754,374,800 in January 2023. …* “ .

**6.)**	doi: <https://doi.org/10.1016/j.najef.2022.101766>; doi:10.1080/07350015.2023.2277172

**Reply:**	Not sure which is the relationship of these two works with the present study. Nevertheless, given that the first deals with the commodity markets we have decided to cite it in the text in the revised version.

**Other minor changes:**

Title page:	Comments from the Editor and two anonymous reviewers are gratefully acknowledged.

We have corrected some other numerous typos and grammatical errors all over the manuscript.

**References**

Bloomfield, P. (1973). An exponential model in the spectrum of a scalar time series, Biometrika 60, 217-226.

Box, G.E.P., Pierce, D.A., 1970. Distribution of residual autocorrelations in autoregressive integrated moving average time series models. J. Am. Stat. Assoc. 65, 1509–1526. <https://doi.org/10.1080/01621459.1970.10481180>.

Breusch, T.S., 1978. Testing for autocorrelation in dynamic linear models. Aust. Econ. Pap. 17, 334–355.

Godfrey, L.G., (1978), Testing against general autoregressive and moving average error models when the regressors include lagged dependent variables. Econometrica 46 (6), 1293–1301.

Ljung, G.M., Box, G.E.P., 1978. On a Measure of Lack of Fit in Time Series Models Biometrika 65 (2), 297–303. McMillan, D.G., Speight, A.E.H., 2001

Zhang, X., X. Yang and Q. He, (2022), Multi-scale systemic risk and spillover networks of commodity markets in the bullish and bearish regimes, The North American Journal of Economics and Finance 62, 101766.
