# Supplementary material for: Average egg price: Mean reversion and persistence in a time series approach
Source: PLoS One. 2026 Mar 25;21(3):e0337054. doi: 10.1371/journal.pone.0337054 (PMC13016358; doi:10.1371/journal.pone.0337054)
Supplement: Attachment — Submitted filename: PLOS ONE Review Report Hassan Tawakol A. Fadol.docx [file pone.0337054.s006.docx]

# Peer Review Report, PLOS ONE

## Manuscript Information

**Title: Average Egg Price: Mean Reversion, Persistence and Structural Breaks**

**Alternative:**

Do Egg Prices Return to Equilibrium? Long-Memory and Structural Dynamics in U.S. Food Markets

**Manuscript ID**: PONE-D-25-28658

**Journal**: PLOS ONE

**Reviewer: Hassan Tawakol Ahmed Fadol, Sudan University of Science and Technology, and ORCID:** [**https://orcid.org/0000-0003-4456-9082**](https://orcid.org/0000-0003-4456-9082)

**Abstract:**

This study investigates the persistence, mean reversion, and structural dynamics of average egg prices in the United States from January 1980 to June 2024. Using fractional integration and the Fractional Cointegrated Vector Autoregressive (FCVAR) model, we examine the long-memory properties of egg prices and their relationship with the Producer Price Index (PPI). Results indicate strong persistence in both series, with fractional differencing parameters between 0.7 and 0.9, implying slow mean reversion rather than strict unit-root behavior. The findings also reveal significant fractional cointegration between egg prices and PPI, confirming a stable long-run equilibrium despite short-term fluctuations. Structural break tests suggest that major disruptions—such as avian influenza outbreaks and pandemic-related shocks—altered the degree of persistence. These results highlight that U.S. egg prices exhibit long-term memory but remain bounded by equilibrium forces, providing relevant insights for policymakers and price stabilization strategies.

**1. Significance**

The paper examines an important and policy-relevant issue — the persistence and mean-reverting behavior of average egg prices in the United States and their co-movement with the Producer Price Index (PPI). Food price stability is a crucial macroeconomic and welfare concern. The topic is both timely and valuable, especially given the high volatility of food prices during recent supply shocks. The application of fractional integration and FCVAR models is appropriate and potentially insightful.

## 2. Originality

## The study’s originality lies in applying fractional integration and FCVAR approaches to egg price dynamics - an area that has received limited econometric attention. However, the contribution would be stronger if the authors explicitly position their study against existing literature on food price persistence that uses ARIMA/SARIMA or volatility models. The paper should better articulate how its methodological approach adds value beyond conventional time-series methods.

## 3. Methodology

**Strengths**

- The use of fractional integration and FCVAR is methodologically consistent with the study’s focus on persistence and long-memory behavior.
- Frequency-domain causality testing (Breitung and Candelon, 2006) is a sound preliminary step before fractional cointegration analysis.

**Major Technical Issues**

1. **Estimation Details Missing:**

The paper does not specify how the differencing parameter *d* was estimated (Whittle, MLE, semiparametric, etc.), nor the software used. Estimation procedures, confidence intervals, and replication details must be clearly stated.

2. **Structural Breaks Not Tested:**

Although the title mentions *Structural Breaks*, no formal test (Bai-Perron, Zivot-Andrews, or breaks in *d*) is presented. This omission undermines the title and conclusions. The authors should either include such tests or revise the title and discussion accordingly.

3. **Seasonality Handling:**

Monthly data are likely to contain seasonal components (e.g., holiday demand). While the paper mentions a seasonal AR(1) model, it does not clarify how seasonality was treated. The authors should discuss the choice of specification and its effect on the persistence parameter.

4. **Residual Diagnostics:**

The results depend on three different noise specifications (white noise, Bloomfield, seasonal AR(1)), but no diagnostic tests (Ljung–Box, ARCH tests, residual plots) are reported. Residual diagnostics are required to justify the final model choice.

5. **Inconsistent Interpretation of *d*:**

At some points, the paper claims “no evidence of short memory (I(0)),” yet elsewhere describes mean reversion (0.5 ≤ *d* < 1). The authors should unify their terminology and interpret *d* consistently throughout.

6. **Data Availability and Transparency:**

Although the data are said to be public, there is no DOI or repository link. Please provide direct data sources (e.g., FRED codes) and, if possible, an open-access CSV and code appendix for reproducibility.

7. **Robustness Checks:**

Consider re-estimating *d* using alternative semiparametric estimators (e.g., GPH, local Whittle) and provide bootstrap confidence intervals to ensure robustness.

## 4. Presentation and Structure

**Language:**

The manuscript is generally clear but would benefit from professional English editing to correct minor grammatical and typographical issues (e.g., inconsistent capitalization and minor spelling errors).

**Tables:**

Tables 2–5 should include the number of observations, AIC/BIC values, and explanatory notes for each column.

**Figures:**

Figures 2–3 need clearer axes labels, time scales, and detailed captions including data sources.

## 5. References

## The references are relevant but could be expanded to include recent applications of fractional cointegration and structural break tests (e.g., Bai & Perron, 2003; Robinson, 1995; Velasco, 1999). Updating these will enhance the paper’s scholarly depth.

**6. Major and Minor Revisions**

**Major Revisions Required**

1. Include **formal structural break tests** (Bai-Perron or Zivot-Andrews) and discuss how breaks affect *d* and cointegration results.
2. Provide **full estimation details** — algorithms, software, sample size, convergence criteria, and replication code if possible.
3. Conduct **robustness checks** using alternative fractional estimators (e.g., GPH, local Whittle) and bootstrap confidence intervals.
4. Re-examine **seasonality** treatment and justify the chosen specification.
5. Add **residual diagnostics** and present key test statistics.
6. Provide **data and code availability** via a repository or appendix.

**Minor Revisions**

Improve figure and table clarity.

Conduct minor language editing.

Clarify the policy implications of findings (what does mean reversion imply for policymakers?).
