# Supplementary material for: Average egg price: Mean reversion and persistence in a time series approach
Source: PLoS One. 2026 Mar 25;21(3):e0337054. doi: 10.1371/journal.pone.0337054 (PMC13016358; doi:10.1371/journal.pone.0337054)
Supplement: Attachment — Submitted filename: new letter addressing the changes.docx [file pone.0337054.s007.docx]

**Plos One**	Prof. Luis A. Gil-Alana

Muhammad Umer Arshad	University of Navarra

Academic Editor	Pamplona, Spain

November 7^th^, 2025

**Ms.Ref. No.:**	PONE-D-25-28658R1

**Title:**	Average Egg Price: Mean reversion, Persistence and Structural Breaks

**Journal:**	PLOS ONE

Dear Editor,

Many thanks for considering our paper entitled “Average Egg Price: Mean reversion,

Persistence and Structural Breaks” as a candidate for publication in Plos One.

Following your advice and the reviewers’ final comments, we have made some

additional changes in the paper that we summarize in the following pages. Hoping this

time the paper might be acceptable for publication in PONE,

Yours sincerely,

Prof. Luis A. Gil-Alana

Yours sincerely

Prof. Luis A. Gil-Alana

University of Navarra

Pamplona

Spain

Reviewers' comments:

Reviewer's Responses to Questions

**Comments to the Author**

**1.)**	If the authors have adequately addressed your comments raised in a previous round of review and you feel that this manuscript is now acceptable for publication, you may indicate that here to bypass the “Comments to the Author” section, enter your conflict of interest statement in the “Confidential to Editor” section, and submit your "Accept" recommendation.

Reviewer #3: All comments have been addressed.

**2.)**	Is the manuscript technically sound, and do the data support the conclusions?

The manuscript must describe a technically sound piece of scientific research with data that supports the conclusions. Experiments must have been conducted rigorously, with appropriate controls, replication, and sample sizes. The conclusions must be drawn appropriately based on the data presented.

Reviewer #3: No. See comments below.

**3.)**	Has the statistical analysis been performed appropriately and rigorously?

Reviewer #3: No. See comments below.

**4.)**	Have the authors made all data underlying the findings in their manuscript fully available?

The [PLOS Data policy](http://track.editorialmanager.com/CL0/http:%2F%2Fwww.plosone.org%2Fstatic%2Fpolicies.action%23sharing/1/010f019a00852394-beae951f-f510-4fe3-be06-894110a7a6ed-000000/kg9I-LysE-BFHaUBw6KhAToec2xiJtu_FZoWxJ9hywo=233) requires authors to make all data underlying the findings described in their manuscript fully available without restriction, with rare exception (please refer to the Data Availability Statement in the manuscript PDF file). The data should be provided as part of the manuscript or its supporting information, or deposited to a public repository. For example, in addition to summary statistics, the data points behind means, medians and variance measures should be available. If there are restrictions on publicly sharing data—e.g. participant privacy or use of data from a third party—those must be specified.

Reviewer #3: No. See comments below.

**5.)**	Is the manuscript presented in an intelligible fashion and written in standard English?

PLOS ONE does not copyedit accepted manuscripts, so the language in submitted articles must be clear, correct, and unambiguous. Any typographical or grammatical errors should be corrected at revision, so please note any specific errors here.

Reviewer #3: Yes	.

**6.)**	Review Comments to the Author

Please use the space provided to explain your answers to the questions above. You may also include additional comments for the author, including concerns about dual publication, research ethics, or publication ethics. (Please upload your review as an attachment if it exceeds 20,000 characters)

**Reviewer #3:**

**1. Significance**
The paper examines an important and policy-relevant issue — the persistence and mean-reverting behavior of average egg prices in the United States and their co-movement with the Producer Price Index (PPI). Food price stability is a crucial macroeconomic and welfare concern. The topic is both timely and valuable, especially given the high volatility of food prices during recent supply shocks. The application of fractional integration and FCVAR models is appropriate and potentially insightful.

Thank you for this positive comment.

**2. Originality**
The study’s originality lies in applying fractional integration and FCVAR approaches to egg price dynamics - an area that has received limited econometric attention. However, the contribution would be stronger if the authors explicitly position their study against existing literature on food price persistence that uses ARIMA/SARIMA or volatility models. The paper should better articulate how its methodological approach adds value beyond conventional time-series methods.

Thanks for this relevant comment. We have added the following paragraph in the literature review section:

“ *… There are numerous studies that have focused the attention in the modelization of food prices throughout different techniques. Thus, for example, Mao et al. (2020) investigated China’s vegetable prices by using ARIMA models in order to determine the factors that influence vegetable price fluctuations. Jayatri (2025) used a more extended ARIMA-GARCH model to examine the volatility in various essential food prices in Indonesia. In a similar vein, Magnogna et al. (2025) employed a hybrid long short-term memory model for the analysis of agricultural price volatility in India. Other studies have performed empirical studies on price forecasting of individual agricultural prices such as soybean (Yin and Wang, 2021), pork (Wang and Sun, 2021; Ye et al., 2021), corn (Xu and Zhang, 2021) and beef (Zeng et al., 2019). Nevertheless, in the economic literature, the statistical properties of the average egg price have received limited attention, …*”.

**References:**

Manogna, R.L., Dharmaji, V. & Sarang, S. (2025), A novel hybrid neural network-based volatility forecasting of agricultural commodity prices: empirical evidence from India. *Journal of Big Data* 12, 85. https://doi.org/10.1186/s40537-025-01131-8

Mao, L, Y. Huang, X. Zhang, S. LI and X. Huang (2020), ARIMA model forecasting analysis of the prices of multiple vegetables under the impact of the COVID-19,  PLoS ONE 17(7): e0271594. https://doi.org/10.1371/journal.pone.0271594

Xu, X.J. and Y. Zhang (2021), Corn cash price forecasting with neural networks. Computers and Electronics in Agriculture,184, 106120

Ye K., Piao, Y.H.R., Zhao, K. and X.H. Cui (2021), A Heterogeneous Graph Enhanced LSTM Network for Hog Price Prediction Using Online Discussion. Agriculture,11(4) 359; <https://doi.org/10.3390/agriculture11040359>.

Yin, T and Y.M. Wang (2021), Market Efficiency and Nonlinear Analysis of Soybean Futures. SustainabilitY13(2), 518,  <https://doi.org/10.3390/su13020518>.

Wang, C.S. and Z.H. Sun (2021),Monthly pork price forecasting method based on Census X12-GM(1,1) combination model.[J]. Plos one,16(5). <https://doi.org/10.1371/journal.pone.0251436>

Zeng, B., S.L. Li, W. Meng and D.H. Zhang (2019), An improved gray prediction model for China’s beef consumption forecasting. Plos one,14(9), pmid:31490952

**3. Methodology**

**Strengths**
• The use of fractional integration and FCVAR is methodologically consistent with the study’s focus on persistence and long-memory behavior.
• Frequency-domain causality testing (Breitung and Candelon, 2006) is a sound preliminary step before fractional cointegration analysis.

Thank you!

**Major Technical Issues
1. Estimation Details Missing:**
The paper does not specify how the differencing parameter d was estimated (Whittle, MLE, semiparametric, etc.), nor the software used. Estimation procedures, confidence intervals, and replication details must be clearly stated.

Thanks for the comment. We have added the following in the methodological section:

“ *… The estimation relies on the parametric approach of Robinson (1994), which is a testing procedure based on the Lagrange Multiplier (LM) principle and that uses the likelihood function in the frequency domain. We use a very simple approach of this method that has been widely used in the empirical literature (see Gil-Alana and Robinson, 1997 for the specific functional form of the tests used in this application, whose codes can be found in Gil-Alana, 1998). The main features of this procedure are …”.*

Also,

**“ …***“ … Data and codes used in this application are available in the following repository 10.5281/zenodo.17415399 at* [*https://zenodo.org/uploads/17415399*](https://zenodo.org/uploads/17415399)*. …”.*

**References**

Gil-Alana, L.A. (1998), Testing fractional integration in macroeconomic time series. PhD thesis, London School of Economics and Political Science, London, UK.

Gil-Alana, L.A. and P.M. Robinson, (1997). Testing of unit root and other nonstationary hypotheses in macroeconomic time series. Journal of Econometrics, 80(2), 241-268. doi:10.1016/s0304-4076(97)00038-9

Robinson, P. M. (1994). Efficient Tests of Nonstationary Hypotheses. Journal of the American Statistical Association, 89(428), 1420–1437. <https://doi.org/10.2307/2291004>

**2. Structural Breaks Not Tested:**
Although the title mentions Structural Breaks, no formal test (Bai-Perron, Zivot-Andrews, or breaks in d) is presented. This omission undermines the title and conclusions. The authors should either include such tests or revise the title and discussion accordingly.

Thanks for letting us know this point. We have proceeded accordingly, removing the presence of “breaks” in the title, and extending a comment in the conclusions about potential lines of future research. The new title is now as follows:

“Average Egg Price: Mean reversion and Persistence in a Time Series Approach”.

We have also added the following comment in the conclusions:

*“ … The paper contains some limitations. First, the possibility of structural breaks is an issue that has not been investigated in the present work. This is relevant since long memory and fractional integration are closely related to the issue of breaks (see, Granger and Hyung, 2008; Barassi et al., 2018; etc.). In this context, the methodology proposed in Gil-Alana (2008), which is basically an extension of Bai and Perron’s (2003) approach to the fractional case can be implemented to the same data used in this application. …”.*

**References**

Bai, J. and P. Perron (2003), Computation and analysis of multiple structural change models, [Journal of Applied Econometrics](https://econpapers.repec.org/article/jaejapmet/) 18, 1, 1-22.

Barassi, M.R., Spagnolo, N. & Zhao, Y. (2018), Fractional Integration Versus Structural Change: Testing the Convergence of  Emissions. *Environ Resource Econ* 71, 923–968. <https://doi.org/10.1007/s10640-017-0190-z>

# Gil-Alana, L.A. (2008), Fractional integration and structural breaks at unknown periods of time, Journal of Time Series Analysis 29, 1, 163-185.

# Granger, C.W.J. and N. Hyung (2004), Occasional structural breaks and long memory with an application to the S&P 500 absolute stock returns, Journal of Empirical Finance  [11, 3](https://www.sciencedirect.com/journal/journal-of-empirical-finance/vol/11/issue/3), 399-421.

**3. Seasonality Handing:**
Monthly data are likely to contain seasonal components (e.g., holiday demand). While the paper mentions a seasonal AR(1) model, it does not clarify how seasonality was treated. The authors should discuss the choice of specification and its effect on the persistence parameter.

This is another relevant point that was not clearly explained in the old manuscript. We have added the following:

*“ … Note that seasonality does not appear as a relevant issue in any of the two series investigated since the estimates of d are practically identical under the seasonal AR specification and the white noise form for the error term. In fact, though unreported, the seasonal AR coefficients were statistically insignificant in the two cases. …”.*

**4. Residual Diagnostics:**
The results depend on three different noise specifications (white noise, Bloomfield, seasonal AR(1)), but no diagnostic tests (Ljung–Box, ARCH tests, residual plots) are reported. Residual diagnostics are required to justify the final model choice.

Thanks for the comment. Note that this cannot be performed in the context of the model of Bloomfield (1973) since this is a non-parametric approach with no explicit functional form. However, performing tests of no autocorrelation in the other two specifications, the results supported the view of no additional time dependence.

*“ … It should finally be noted that several Ljung-Box Q-test statistics were conducted on the residuals from the white noise and seasonal AR(1) specifications and the results suggested that they were no additional serial correlation in the data. …”.*

**References**

Bloomfield, P. (1973). An exponential model in the spectrum of a scalar time series, Biometrika 60, 217-226.

**5. Inconsistent Interpretation of d:**
At some points, the paper claims “no evidence of short memory (I(0)),” yet elsewhere describes mean reversion (0.5 ≤ d < 1). The authors should unify their terminology and interpret d consistently throughout.

Thanks for the comment. We refer to short memory or I(0) behaviour if the hypothesis of d = 0 is rejected in the data. This happens if that value does not belong to the 95% confidence intervals reported in the tables. Mean reversion takes place if d is smaller than 1. We have clarified these points all over the manuscript.

In this context, we have changed two sentences in the Abstract, that reads now as follows:

*“This study investigates the long-term behavior of average egg prices in the U.S., using fractional integration techniques. The analysis also explores the relationship between egg prices and the Producer Price Index (PPI) through cointegration methods. The findings suggest long memory in both series and mean reversion for PPI. Additionally, the FCVAR model indicates a potential long-run equilibrium relationship between average egg prices and the PPI.”*.

Also, in the conclusions we have made this point much more explicit:

*“ … The primary discovery of this paper supports the notion that disturbances in average egg prices will have enduring effects in the U.S., as none of the series displays short memory traits (i.e., I(0)). Thus, long memory takes place in all cases with positive values of d across all confidence intervals. This suggests that abrupt changes or fluctuations in prices are likely to have a more prolonged impact. Moreover, the fact that the intervals include the value of d = 1 indicates that we cannot reject the hypothesis of a unit root in the series and therefore we may reject the hypothesis of reversion to the mean. On the other hand, regarding the Producer Price Index, despite the absence of short memory traits, the parameter d falls within the range 0.5 ≤ d < 1. This indicates that the time series is nonstationary with mean reversion,*

**6. Data Availability and Transparency:**
Although the data are said to be public, there is no DOI or repository link. Please provide direct data sources (e.g., FRED codes) and, if possible, an open-access CSV and code appendix for reproducibility.

We have added the following comment:

“ … *We use a very simple approach of this method that has been widely used in the empirical literature (see Gil-Alana and Robinson, 1997 for the specific functional form of the tests used in this application, whose codes can be found in Gil-Alana, 1998). …”.*

Also, in the data section:

*“ … Data and codes used in this application are available in the following repository 10.5281/zenodo.17415399 at* [*https://zenodo.org/uploads/17415399*](https://zenodo.org/uploads/17415399)*. …”.*

**References**

Gil-Alana, L.A. (1998), Testing fractional integration in macroeconomic time series. PhD thesis, London School of Economics and Political Science, London, UK.

Robinson, P. M. (1994). Efficient Tests of Nonstationary Hypotheses. Journal of the American Statistical Association, 89(428), 1420–1437. <https://doi.org/10.2307/2291004>

**7. Robustness Checks:**
Consider re-estimating d using alternative semiparametric estimators (e.g., GPH, local Whittle) and provide bootstrap confidence intervals to ensure robustness.

Thanks for this comment. We have included the following comment at the end of the Methodological section:

*“ … Nevertheless, as a robustness check, we also employed alternative approaches like the parametric method of Sowell (1992) and the semiparametric log-periodogram estimate of Geweke and Porter-Hudak (1983) and others like Robinson (1995) and Velasco (1999), all producing very similar results to those reported in this work. …”.*

**References**

Geweke, J. & Porter-Hudak, S. (1983), The estimation and application of long memory time series. *Journal of Time Series Analysis 4*(4), 221-238. https://doi.org/10.1111/j.1467-9892.1983.tb00371.x

Robinson, P. M. (1995), Gaussian semiparametric estimation of long range dependence, Annals of Statistics 23, 1630-1661.

Sowell, F. (1992), Maximum likelihood estimation of stationary univariate fractionally integrated time series models, *Journal of Econometrics 53*(1-3), 165-188. https://doi.org/10.1016/0304-4076(92)90084-5

# Velasco, C. (1999), Gaussian Semiparametric Estimation of Non-stationary Time Series, Journal of Time Series Analysis 20, 1, 87-127.

**8. Presentation and Structure
Language:**
The manuscript is generally clear but would benefit from professional English editing to correct minor grammatical and typographical issues (e.g., inconsistent capitalization and minor spelling errors).

Thanks. The paper has been sent to an English native speaker colleague for revision.

**Tables:**
Tables 2–5 should include the number of observations, AIC/BIC values, and explanatory notes for each column.

Endnotes have been incorporated across the tables.

**Figures:**
Figures 2–3 need clearer axes labels, time scales, and detailed captions including data sources.

Done.

**5. References**
The references are relevant but could be expanded to include recent applications of fractional cointegration and structural break tests (e.g., Bai & Perron, 2003; Robinson, 1995; Velasco, 1999). Updating these will enhance the paper’s scholarly depth.

The following references have been added in the new version of the manuscript.

**References**

Bai, J. and P. Perron (2003), Computation and analysis of multiple structural change models, [Journal of Applied Econometrics](https://econpapers.repec.org/article/jaejapmet/) 18, 1, 1-22

Barassi, M.R., Spagnolo, N. & Zhao, Y. (2018), Fractional Integration Versus Structural Change: Testing the Convergence of  Emissions. *Environ Resource Econ* 71, 923–968. <https://doi.org/10.1007/s10640-017-0190-z>

Gil-Alana, L.A. (1998), Testing fractional integration in macroeconomic time series. PhD thesis, London School of Economics and Political Science, London, UK.

# Gil-Alana, L.A. (2008), Fractional integration and structural breaks at unknown periods of time, Journal of Time Series Analysis 29, 1, 163-185.

# Granger, C.W.J. and N. Hyung (2004), Occasional structural breaks and long memory with an application to the S&P 500 absolute stock returns, Journal of Empirical Finance  [11, 3](https://www.sciencedirect.com/journal/journal-of-empirical-finance/vol/11/issue/3), 399-421.

Jayatri, U.F. (2025), Modelling and Predicting Volatility in Essential Food Prices Using ARIMA-GARCH Models, Effective Efficient: Journal of Industrial Engineering 1, 1.

Manogna, R.L., Dharmaji, V. & Sarang, S. (2025), A novel hybrid neural network-based volatility forecasting of agricultural commodity prices: empirical evidence from India. *Journal of Big Data* 12, 85. https://doi.org/10.1186/s40537-025-01131-8

Mao, L, Y. Huang, X. Zhang, S. LI and X. Huang (2020), ARIMA model forecasting analysis of the prices of multiple vegetables under the impact of the COVID-19,  PLoS ONE 17(7): e0271594. https://doi.org/10.1371/journal.pone.0271594

Robinson, P. M. (1995), Gaussian semiparametric estimation of long range dependence, Annals of Statistics 23, 1630-1661.

Sowell, F. (1992), Maximum likelihood estimation of stationary univariate fractionally integrated time series models, *Journal of Econometrics 53*(1-3), 165-188. https://doi.org/10.1016/0304-4076(92)90084-5

# Velasco, C. (1999), Gaussian Semiparametric Estimation of Non-stationary Time Series, Journal of Time Series Analysis 20, 1, 87-127.

Xu, X.J. and Y. Zhang (2021), Corn cash price forecasting with neural networks. Computers and Electronics in Agriculture,184, 106120

Ye K., Piao, Y.H.R., Zhao, K. and X.H. Cui (2021), A Heterogeneous Graph Enhanced LSTM Network for Hog Price Prediction Using Online Discussion. Agriculture,11(4) 359; <https://doi.org/10.3390/agriculture11040359>.

Yin, T and Y.M. Wang (2021), Market Efficiency and Nonlinear Analysis of Soybean Futures. SustainabilitY13(2), 518,  <https://doi.org/10.3390/su13020518>.

Wang, C.S. and Z.H. Sun (2021),Monthly pork price forecasting method based on Census X12-GM(1,1) combination model.[J]. Plos one,16(5). <https://doi.org/10.1371/journal.pone.0251436>

Zeng, B., S.L. Li, W. Meng and D.H. Zhang (2019), An improved gray prediction model for China’s beef consumption forecasting. Plos one,14(9), pmid:31490952
